# *In vitro* and *in vivo* approaches to assess atherosclerosis following exposure to low-dose mixtures of arsenic and cadmium

**DOI:** 10.1016/j.taap.2023.116763

**Published:** 2023-11-18

**Authors:** Nivetha K. Subramaniam, Natascha Gagnon, Kiran Makhani, Nikola Kukolj, Michael H. Mouradian, Braeden H. Giles, Harinee Srikannan, Victoria Fruh, Jaymie Meliker, Gregory A. Wellenius, Koren K. Mann

**Affiliations:** a Division of Experimental Medicine, McGill University, Montreal, Quebec, Canada; b Department of Pharmacology and Therapeutics, McGill University, Montreal, Quebec, Canada; c Lady Davis Institute for Medical Research, Jewish General Hospital, Montreal, Quebec, Canada; d Department of Environmental Health, Boston University School of Public Health, Boston, MA, USA; e Program in Public Health, Department of Family, Population, & Preventive Medicine, Stony Brook University, Stony Brook, NY, USA; f Center for Climate and Health, Boston University School of Public Health, Boston, MA, USA

**Keywords:** Arsenic, Cadmium, Low-Dose, Mixtures, Atherosclerosis

## Abstract

Worldwide, millions of people are co-exposed to arsenic and cadmium. Environmental exposure to both metals is linked with a higher risk of atherosclerosis. While studies have characterized the pro-atherosclerotic effects of arsenic and cadmium as single agents, little is known about the potential effects of metal mixtures, particularly at low doses. Here, we used a combination of *in vitro* and *in vivo* models to assess the effects of low-dose metals individually and as mixtures on early events and plaque development associated with atherosclerosis. *In vitro*, we investigated early pro-atherogenic changes in macrophages and endothelial cells with metal treatments. The combined cytotoxic effects of both metals at low concentrations were dose interactive, specifically, synergistic in macrophages, but antagonistic in endothelial cells. Despite this differential behavior across cell types, the mixtures did not initiate early pro-atherogenic events: neither reactive oxygen species generation in macrophages nor adhesion molecule expression on endothelial cells. *In vivo*, we utilized the well-characterized hyperlipidemic apolipoprotein E knock-out (ApoE^−/−^) mouse model. Previously, we have shown that low concentrations of arsenic (down to 10 ppb) enhance atherosclerosis in ApoE^−/−^mice. This model has also been used with cadmium to demonstrate pro-atherogenic effects, although at concentrations above human-relevant exposures. In both sexes, there are some small increases in atherosclerotic lesion size, but very few changes in plaque constituents in the ApoE^−/−^mouse model. Together, these results suggests that low-dose metal mixtures are not significantly more pro-atherogenic than either metal alone.

## 1. Introduction

Environmental exposure to metals, such as arsenic (As) and cadmium (Cd), have been associated with higher risk of cardiovascular diseases, including atherosclerosis (Lamas et al., 2023). Atherosclerosis is a vascular disease characterized by the buildup of fibro-fatty plaque in the inner lining of an artery. Atherosclerotic plaque development begins with the perturbation of endothelial function (Gimbrone Jr. et al., 2000) by excess oxidized lipid followed by monocyte/macrophage recruitment, infiltration and differentiation. These myeloid cells then engulf the oxidized lipid, forming foam cells, and initiate an inflammatory cascade (Moore and Tabas, 2011). This leads to the first stage in plaque development, the formation of a fatty streak (Douglas and Channon, 2014). The continued recruitment of leukocytes further promotes lipoprotein retention, extracellular matrix alteration and sustained chronic inflammation. As the atherosclerotic lesion progresses, vascular smooth muscle cells (VSMCs) migrate, proliferate to secrete extracellular matrix and form a protective fibrous cap (Harman and Jørgensen, 2019). In advanced stages, the apoptosis and defective efferocytosis of macrophages (Tabas, 2010) and VSMCs (Harman and Jørgensen, 2019) contribute to plaque necrosis. Plaques with large necrotic cores and thin fibrous caps are prone to rupture and cause myocardial infarction (Virmani et al., 2006).

Millions of people are exposed to arsenic and cadmium worldwide. Although combined metal exposure is derived from multiple sources, one of the primary sources of arsenic exposure is contaminated drinking water (Chung et al., 2014). The World Health Organization (WHO) has set the maximum contaminant level of arsenic in drinking water at 10 ppb (World Health, 2019). Nevertheless, higher arsenic levels have been reported in Brazil (Mirlean et al., 2014), Bangladesh (Chakraborti et al., 2010), India (Shukla et al., 2010), Cambodia (Sthiannopkao et al., 2008), Chile (Nriagu et al., 2007), Spain (Gómez et al., 2006), Thailand (Santha et al., 2022) and United States (Naujokas et al., 2013). Cadmium is a metal with a long biological half-life in the human body ranging from 15 to 30 years (Rafati Rahimzadeh et al., 2017). The main sources of Cd exposure for non-smoking and non-occupationally exposed individuals, are diet, air and groundwater (Kubier et al., 2019; Tinkov et al., 2018). The WHO set the maximum contaminant level of Cd in drinking water at 3 ppb (World Health, 2004). In general, low levels (0.2 to 2 ppb) of Cd have been reported in groundwater, for instance, in the western part of United States (Ayotte et al., 2011) and Northern Germany (Rodemann et al., 1983). However, there are some regions where levels greater than the WHO-set limit of Cd have been reported. For example, some waste sites in the United States have greater levels, as a result of both natural and anthropogenic processes (Kubier et al., 2019). Furthermore, arsenic and Cd are often found together in mines, agricultural systems, and industrial wastewater discharges (Gunadasa et al., 2023; Arancibia-Miranda et al., 2020). More importantly, both metals are reported to co-contaminate drinking water supply systems (Gao et al., 2019; Sierra-Sánchez et al., 2022; Oseghe et al., 2021; Zhou et al., 2023). This, combined with exposure from other sources, means that exposure to low levels of metal mixture is profound.

Metals can affect multiple points along the pathogenesis of atherosclerosis (Grau-Perez et al., 2022). Both arsenic and Cd are pro-atherogenic. Mechanistic evidence suggests that arsenic induces endothelial dysfunction, increases reactive oxygen species, impairs nitric oxide balance, and amongst other pro-atherogenic properties, all of which enhance atherogenesis (States et al., 2009). On a similar note, Cd targets the vascular endothelium, induces endothelial dysfunction *via* modulation of adhesion molecules (Prozialeck et al., 2006) and reduces nitric oxide bioavailability (Kolluru et al., 2006). Moreover, both arsenic and Cd enhance atherosclerosis in the well-characterized, hyperlipidemic apolipoprotein E knock-out (ApoE^−/−^) mouse model as single agents. We have previously shown that low concentrations of arsenic (down to 10 ppb) induce atherosclerosis in ApoE^−/−^mice (Makhani et al., 2018). In addition, Cd has pro-atherogenic effects in ApoE^−/−^mice, although at concentrations above human-relevant exposures (Oliveira et al., 2019). Thus, both arsenic and Cd can enhance the development of atherosclerosis.

While studies have characterized the effects of arsenic and Cd as single agents, many of these studies, particularly for Cd, have utilized concentrations well above environmentally-relevant doses. Moreover, these studies have not characterized the effects of mixtures of metals, more closely mimicking the complex environments we are all exposed to. Here, we used a combination of *in vitro* and *in vivo* approaches to study the effects of low-dose arsenic/Cd as single agents and as mixtures on early events and plaque development associated with atherosclerosis. *In vitro*, we investigated early pro-atherogenic changes in macrophages and endothelial cells with metal treatments. *In vivo*, we utilized low-dose, environmentally-relevant concentrations of arsenic, Cd and the co-exposure in the ApoE^−/−^mice and considered sex as a biological variable.

## 2. Materials and methods

### 2.1. Cell viability assay

RAW 264.7 (ATCC-TIB-71) murine macrophages and C166 (ATCC CRL-2581) murine endothelial cells were routinely cultured in Dulbecco’s Modified Eagle Medium (DMEM) media at 37 °C in 5% CO_2_. The range of concentrations chosen for each metal are as follows: 10 nM – 5μM arsenic and 10 nM – 10 *μ*M Cd, ranges encompassing both low environmental and higher *in vitro* concentrations. Arsenic, cadmium and mixture working solutions were prepared from sodium arsenite and cadmium chloride stock solutions, respectively, and solubilized with deionized water. The cells were seeded in a 96-well plate and cultured with the metal treatments for 24 h. The cytotoxicity in each cell type were determined using the CellTiter-Glo Luminescent Cell Viability Assay. CellTiter-Glo staining measures the number of viable cells in culture based on quantitation of the ATP present, an indicator of metabolically active cells. Synergy was assessed by excess over bliss (EOB) analysis (Borisy et al., 2003).

### 2.2. High-content imaging

RAW 264.7 cells were seeded in PhenoPlate 96-well microplates (Perkin Elmer) coated with 10 μg/ml poly-d-lysine (ThermoFisher) at a seeding density of 4000 cells/well. The cells were exposed to arsenic and cadmium treatments for 24 h. Menadione was used as a positive control. Following the metal exposures, the RAW 264.7 cells were treated with cell permeable fluorescent dyes for live-cell imaging. These dyes include CellROX Deep Red reagent (ThermoFisher) for reactive oxygen species detection, CellMask Green (ThermoFisher) for plasma membrane stain and Hoechst (ThermoFisher) for nuclear stain. Cells were stained at 37 °C in 5% CO_2_ for 30 min. The Operetta High-Content Imaging System (Perkin Elmer) was used for live-cell imaging (9 fields/well, 4 planes/well, 40× magnification). The images were then analyzed using the Columbus Image Data Storage and Analysis System (Perkin Elmer).

### 2.3. Flow cytometry

C166 (ATCC CRL-2581) murine endothelial cells were seeded in a 12-well plate at a seeding density of 100 000 cells/well and treated with arsenic and/or cadmium for 4 h or 24 h. Lipopolysaccharide (LPS) was used as the positive control. Post treatment, cells were harvested with trypsin and washed with 1× phosphate buffered saline. Cells were then stained with anti-mouse CD106 (VCAM-1) (ThermoFisher) and Near-IR Live/Dead (ThermoFisher). The BD FACSCanto II Flow Cytometer (Lady Davis Institute Flow Cytometry Core) was used to measure the mean fluorescence intensity of VCAM-1 positive cells. FlowJo v10 was used for analysis.

### 2.4. Incucyte live-cell analysis system

RAW 264.7 cells were seeded in PhenoPlate 96-well microplates (Perkin Elmer) at a seeding density of 3000 cells/well. The cells were exposed to arsenic and cadmium treatments for 24 h on day 2. GW3965, a synthetic LXR agonist, was used as a control. Following the metal exposures, cells were incubated with 2.5 μg/ml red-orange Dil dye-labeled oxLDL (Dil-oxLDL) (ThermoFisher) for 4 h. The cells were imaged in the S3 Incucyte Imaging System (Sartorius) and the mean Dil-OxLDL intensity was recorded every 30 min in real time.

### 2.5. Enzyme-linked immunosorbent assay (ELISA)

RAW 264.7 macrophages were seeded in a 6 well plate at a seeding density of 200 000 cells/well. The cells were treated with arsenic and cadmium treatments for 24 h on day 2. LPS was used as a positive control. Following the metal exposure, the supernatant was collected and snap frozen. TNF-α, IL-1β and IL-6 were quantified in the conditioned media using R&D DuoSet ELISAs for TNF-α (DY410–05), IL-1β (DY401–05), and IL-6 (DY406–05). Optical density (OD) values were calculated by subtracting the OD 540 nm from the OD 450 nm.

### 2.6. In vivo arsenic and cadmium exposure

All mouse studies were approved by the McGill Animal Care and Use Committee. ApoE^−/−^mice (B6.129P2—apoEtm1Unc/J from Jackson Laboratory) were bred in the Lady Davis Institute Animal Facility. Starting at 5 weeks of age, male and female ApoE^−/−^mice were given low arsenic/cadmium chow (AIN-76; Envigo, Lachine, Quebec). Mice were exposed to tap water sodium arsenite (5 or 50 ppb arsenic), cadmium chloride (1.5 or 5 ppb cadmium), or the combinations for 13 weeks. Arsenic/cadmium drinking water was changed three times per week. Body weight was monitored. At endpoint, mice were euthanized with isoflurane, and the aortas and hearts were dissected.

### 2.7. Atherosclerotic lesion characterization

The size and characterization of the atherosclerotic lesions in the aorta and aortic sinus were assessed as previously described (Lemaire et al., 2011; Makhani et al., 2018). In short, the fixed aorta was rinsed with ultrapure water, then cut longitudinally and stained *en face* with oil red O (Electronic Microscopy Sciences) for triglycerides, lipids and cholesterol. Images were obtained using INFINITYCAPTURE software and camera (Lumenera). The percentage of lesion area of the aortic arch, defined as the region from the first intercostal arteries to the ascending arch, were evaluated using ImageJ software [National Institutes of Health (NIH)]. The atherosclerotic lesions were also evaluated within the aortic sinus. A total of 8 animals were used for both analyses for each treatment and sex. The hearts were rinsed, fixed, embedded, and processed as previously described (Lemaire et al., 2011; Makhani et al., 2018). Approximately 7 μm cryosections were sliced from the aortic base throughout the aortic sinus, and consecutive sections were collected on 10 different slides. On average, 4–9 slices were collected on each slide, and each slice was about 70 μm apart. Individual slides were stained with oil red O to determine the plaque areas and their lipid content. The aortic sinuses were also stained and analyzed for collagen content (type I and type III) using picrosirius red (Polysciences).

### 2.8. In situ immunofluorescence

Smooth muscle cell (SMC) and macrophage content were assessed within the plaque area as previously described (Lemaire et al., 2011; Makhani et al., 2018). In short, aortic sinus sections were rinsed and blocked with 3% bovine serum albumin (Sigma-Aldrich), incubated with primary antibody [1:100 for monoclonal anti-α-smooth muscle cell actin [clone 1A4], 1:50 for MOMA-2 (macrophages) antibody (Abcam)], rinsed, and incubated with fluorescently labeled secondary antibodies (1:500; Invitrogen). The immunofluorescent marker from at least 4–9 sections per animal were quantified using ImageJ software (NIH) and expressed as a percentage of total lesion area.

### 2.9. Plasma analyses

Blood (0.6 mL) was collected by cardiac puncture and plasma was obtained using collection tubes (ethylenediaminetetraacetic acid BD Vacutainer SST). Cholesterol, high-(HDL) and low-density (LDL) lipoproteins, triglycerides, and glucose were assessed by the pathology services at The Centre of Phenogenomics in Toronto, Ontario.

### 2.10. Statistical analyses

Statistical analyses were performed using GraphPad software (La Jolla, California) using a one-way ANOVA and a two-way ANOVA with a Tukey’s multiple comparisons test. A two-sided *p* <0. 05 was considered statistically significant. Statistical test is indicated in figure legends where applicable.

## 3. Results

### 3.1. In vitro assessment of early proatherogenic changes following low dose As/Cd mixtures

**Fig. 1.**
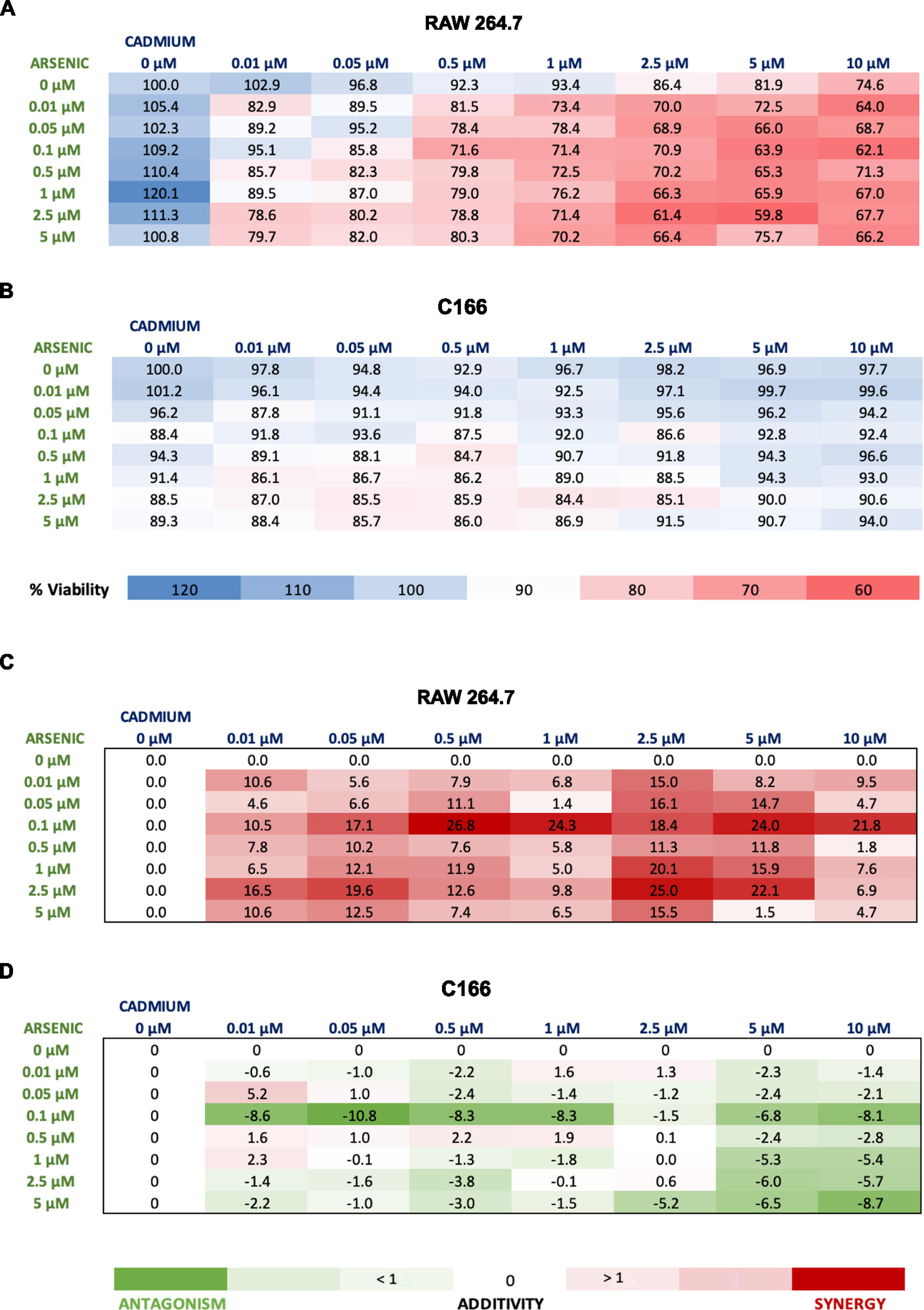
Differential sensitivity to low-dose metal mixtures in RAW 264.7 macrophages and C166 endothelial cells. The combined effect of both metals is synergistic in RAW 264.7 cells whereas antagonistic in C166 cells. The mean percent cell viability (*n* = 3) in RAW 264.7 cells (A) and C166 cells (B) after arsenic and cadmium treatments for 24 h. Cell viability was measured as a change in CellTiter-Glo luminescent signal, blue regions correspond to increased cell viability whereas red regions correspond to decreased cell viability. Mean excess over bliss analysis (n = 3) in RAW 264.7 cells (C) and C166 cells (D), red regions illustrate synergy amongst arsenic and cadmium whereas green regions illustrate antagonism. No statistical differences were observed in cell viability and in excess over bliss in both cell lines. (For interpretation of the references to colour in this figure legend, the reader is referred to the web version of this article.)

Previous data indicate that both arsenic and Cd have pro-atherogenic properties, however, it is unclear whether these extend to low concentrations and/or to the mixture of the two metals. We used *in vitro* approaches to study the effects of exposure to mixtures of arsenic and Cd and focused on early events in atherogenesis: reactive oxygen species (ROS) generation in macrophages (Xu et al., 2019), the expression of adhesion molecules on endothelial cells (Xu et al., 2019; Gimbrone Jr. and García-Cardeña, 2016; Insull Jr., 2009), oxidized lipoprotein uptake in macrophages and release of cytokines (Crowther, 2005; Gaggini et al., 2023). First, we determined the cell viability following metal exposure, for single metal exposures or for combined metal exposures, in either RAW 264.7 murine macrophages or C166 endothelial cells after 24 h. We tested a broad range of concentrations for both metals from 0.01 to 5 μM arsenic and 0.01–10 μM Cd. In RAW macrophages, Cd exposure singularly resulted in a dose-dependent decrease in viability, while, interestingly, arsenic exposure singularly increased viability until 1 μM (Fig. 1A & Table S1). The combination of metals resulted in a dose-dependent increase in cytotoxicity reaching a maximum of ~60% viability. In the C166 endothelial cells, each metal alone resulted in a dose-dependent decrease in viability with arsenic being more cytotoxic (Fig. 1B & Table S1). The As/Cd combinations decreased cell viability, although not more than ~15%, indicating that the endothelial cells are more resistant to the cytotoxic effects of arsenic and Cd, individually and together. The excess over bliss (EOB) independence model (Bansal et al., 2014) was used to determine synergy/antagonism between arsenic and Cd in both the RAW 264.7 cells (Fig. 1C & Table S2) and the C166 cells (Fig. 1D & Table S2). A compound pair with positive EOB values depicts synergistic behavior, while negative EOB values depict antagonistic behavior, and an EOB value of 0 corresponds to an additive effect. Interestingly, here, mixtures of low-dose arsenic and Cd behave differently in macrophages than in endothelial cells. The effect of both metals combined at low concentrations appears to be dose interactive, specifically, synergistic in the RAW cells (Fig. 1C) but antagonistic in the C166 cells (Fig. 1D). However, in both cell lines, no statistically significant differences were found in cell viability and in EOB using a one-way ANOVA with a Tukey’s post-hoc test after low-dose metal treatments for 24 h. To reduce the confounding cytotoxicity in downstream analyses, we focused on concentration mixtures that yielded >75% viability.

**Fig. 2.**
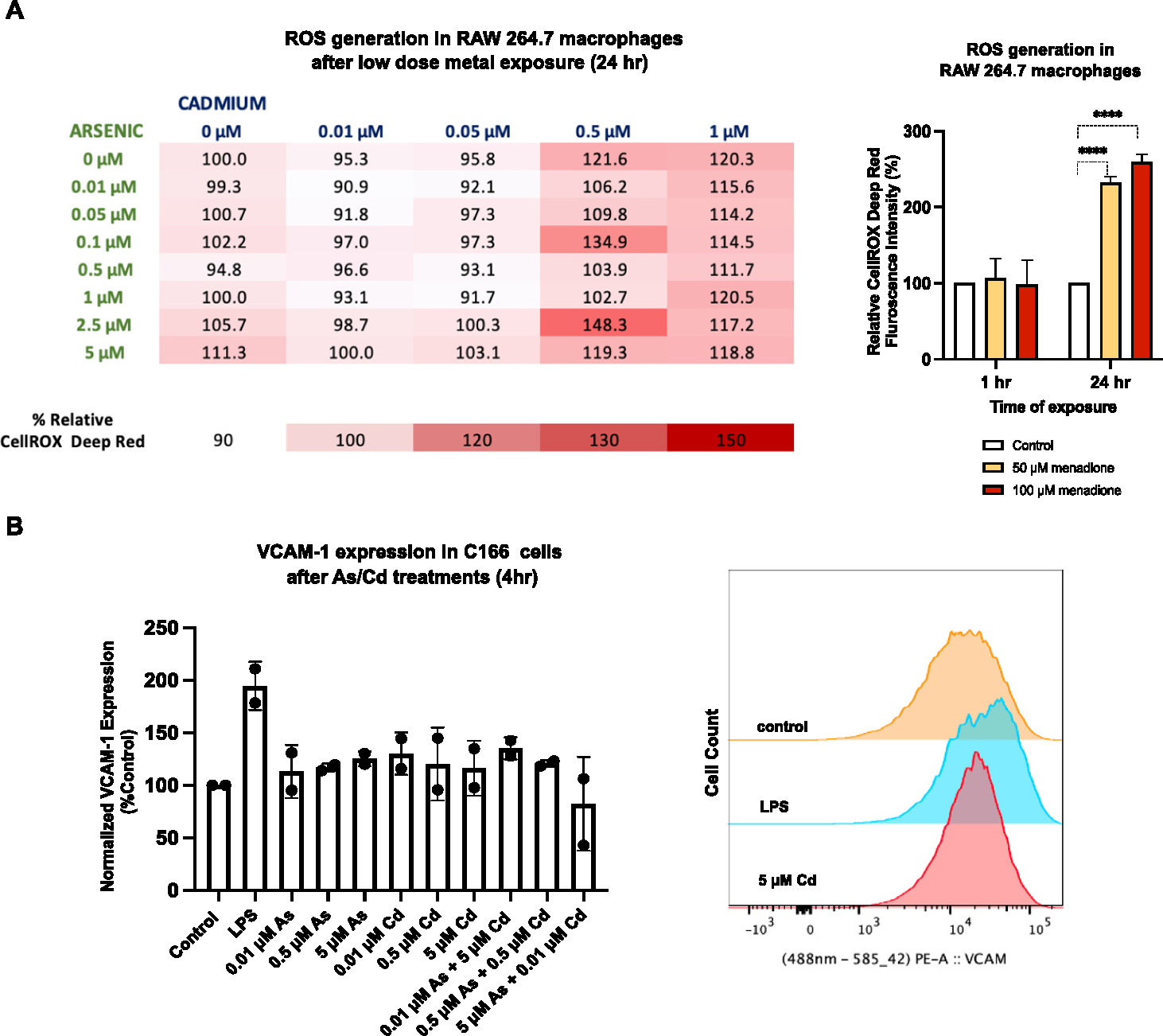

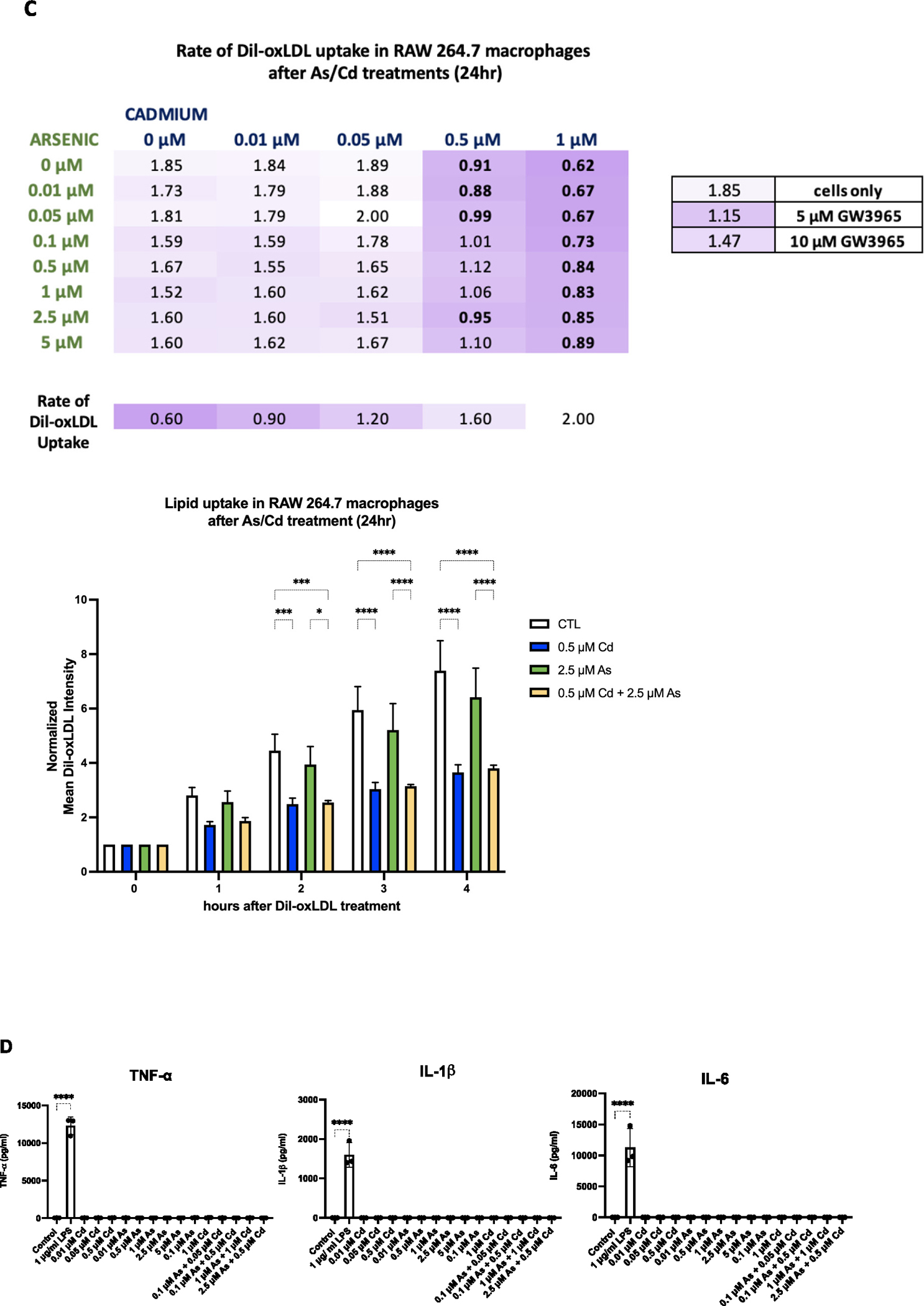
*In vitro* approaches to study atherosclerosis following exposure to low-dose arsenic and cadmium. (A) Intracellular ROS levels were measured and quantified as a change in CellROX Deep Red fluorescence signal by high content screening. Increased ROS levels are seen at higher concentrations of arsenic, cadmium and in combinations, although not statistically significant. Menadione was used as a positive control. Statistical significance is represented as follows: ****p < .0001. (B) Flow cytometry analysis of the expression of VCAM-1 on C166 cells under low dose-arsenic and cadmium treatment over 4 h (*n* = 2). No changes were observed in VCAM-1 expression after low-dose metal treatments. (C) Dil-oxLDL uptake was measured in RAW cells after 24-h metal exposure (*n* = 3). At higher concentrations of cadmium, the rate of Dil-oxLDL uptake (per hour) was significantly reduced (represented in bold) singularly and in combinations compared to cells only. A two-way ANOVA with a Tukey’s multiple comparison test was done. Statistical significance is represented as follows: *p < .05, **p < .01, ***p < .001 and ****p < .0001. (D) No change in TNF-α, IL-1β and IL-6 expression after exposure to arsenic and cadmium in RAW 264.7 cells. LPS was used as a positive control. (For interpretation of the references to colour in this figure legend, the reader is referred to the web version of this article.)

As a first approach to study pro-atherogenic mechanisms, we measured the levels of ROS in macrophages after treatment with metal mixtures. We cultured RAW 264.7 murine macrophages with the experimentally determined non-cytotoxic concentrations of arsenic and Cd for 24 h and assessed superoxide production by high content imaging. Menadione was used as a positive control, where the ROS level significantly increased in the RAW 264.7 cells after 24 h exposure with menadione but not 1 h (Fig. 2A). With higher concentrations of arsenic, Cd and in combinations, there is an increase in ROS, although not statistically significant (*p* = .069–0.6501). Next, we measured VCAM-1 expression in C166 cells after low-dose metal treatments at 4 h and 24 h using flow cytometry. LPS was used as a positive control, which increased VCAM-1 expression after 4 h. However, at neither time point was VCAM-1 expression changed after arsenic and Cd exposure (Fig. 2B & S1). Then, we measured Dil-oxLDL uptake in RAW 264.7 cells after 24 h exposure to arsenic, Cd and in combinations over time (Fig. 2C). There was no change in the rate of lipid uptake (per hour) with arsenic treatment. However, Cd exposure (0.5 μM and 1 μM Cd) significantly decreased the rate of lipid updake alone and in some of the combinations compared to controls or arsenic. More interestingly, in some of the combinations, the addition of arsenic to 0.5 μM or 1 μM Cd did not alter the decreased rate of lipid uptake compared to Cd alone (Fig. 2C & Table S3). As an indication for vascular inflammation, we also measured cytokines promoting atherogenesis, TNF-α, IL-1β and IL-6 (Tousoulis et al., 2016) in conditioned media after metal exposure to RAW 264.7 cells. There was no change in cytokine expression with arsenic, Cd, or the combinations, although the positive control (LPS) consistently induced cytokine production (Fig. 2D). While these data suggest that low-dose mixtures of arsenic/Cd did not initiate pro-atherogenic events, atherosclerosis is a complex, multi-factorial process and as such, we extended our analysis to an *in vivo* model.

### 3.2. In vivo assessment of plaque size in ApoE^−/−^mice exposed to low concentrations of as/cd mixtures

We have previously shown that in the ApoE^−/−^mouse model low to moderate levels of arsenic, 10–200 ppb (Makhani et al., 2018), increases the size of the atherosclerotic lesion in a dose-dependent manner. In addition, pro-atherogenic effects have been reported in the same model treated with 100 ppm Cd (Oliveira et al., 2019). Here, we utilized this well-characterized *in vivo* model of atherosclerosis to study low-dose, environmentally relevant exposures of arsenic and Cd as individual metals and as mixtures. We exposed both male and female ApoE^−/−^mice to tap water, arsenic (5 or 50 ppb), Cd (1.5 or 5 ppb), or the combinations for 13 weeks. This is a time point at which we have previously observed arsenic-enhanced plaque size (Makhani et al., 2018). At endpoint, we determined the size of atherosclerotic lesions in the aortic arch by *en face* oil red O staining on the luminal side of the aorta.

**Fig. 3.**
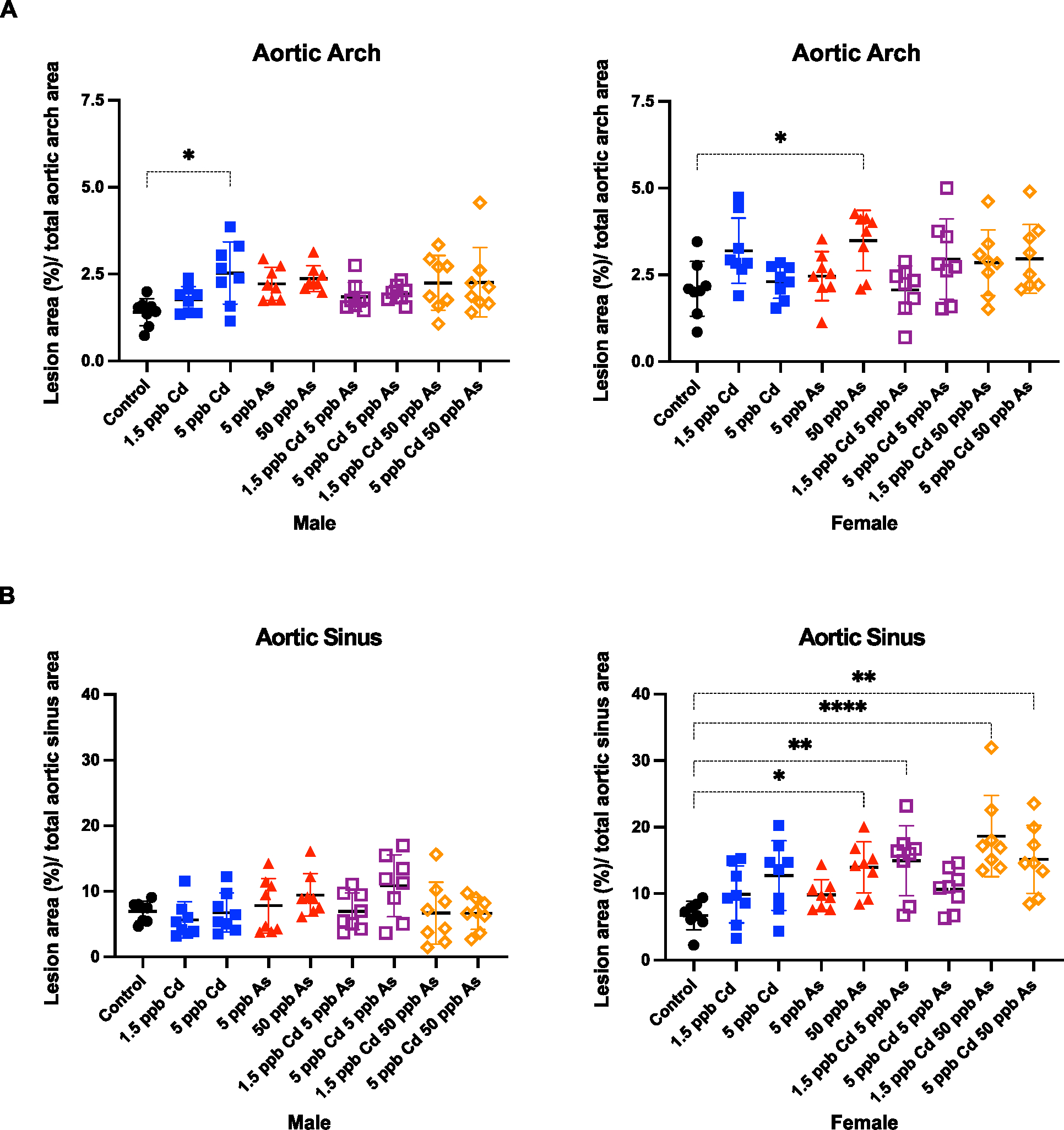
Sex-specific differences in the size of atherosclerotic lesion in the aortic arch (A) and aortic sinus (B) after low-dose arsenic, cadmium, and co-exposure in ApoE^−/−^mice. All the plaques were smaller than those observed with higher concentrations of arsenic and/or high-fat diet. Plaque was quantified after oil red O staining and imaged using ImageJ. Each point represents a single mouse. Statistical significance is represented as follows: *p < .05; **p < .01; ****p < .0001. (For interpretation of the references to colour in this figure legend, the reader is referred to the web version of this article.)

In both sexes, there were some increases in the lesion size in the aortic arch (Fig. 3A). In males, 5 ppb Cd increased the lesion size significantly in the aortic arch (Fig. 3A, p < 0.05), while arsenic alone resulted in no statistically significant changes. On the other hand, in females, 50 ppb arsenic significantly increased the lesion size in the aortic arch (Fig. 3A, p < 0.05), which we have previously reported in males (Makhani et al., 2018). Of note, the metal combinations did not increase arch plaque size significantly in either males or females. Notably, in males, arsenic, Cd and/or the combinations of both metals did not significantly increase the size of the atherosclerotic plaque in the aortic sinus. On the contrary, in females, 50 ppb arsenic and some of the combinations significantly increased the lesion size in the aortic sinus (Fig. 3B, p < 0.05; *p* < .01; p < .0001).

### 3.3. Assessment of plaque components following exposure to low concentrations of arsenic/cd mixtures

**Fig. 4.**
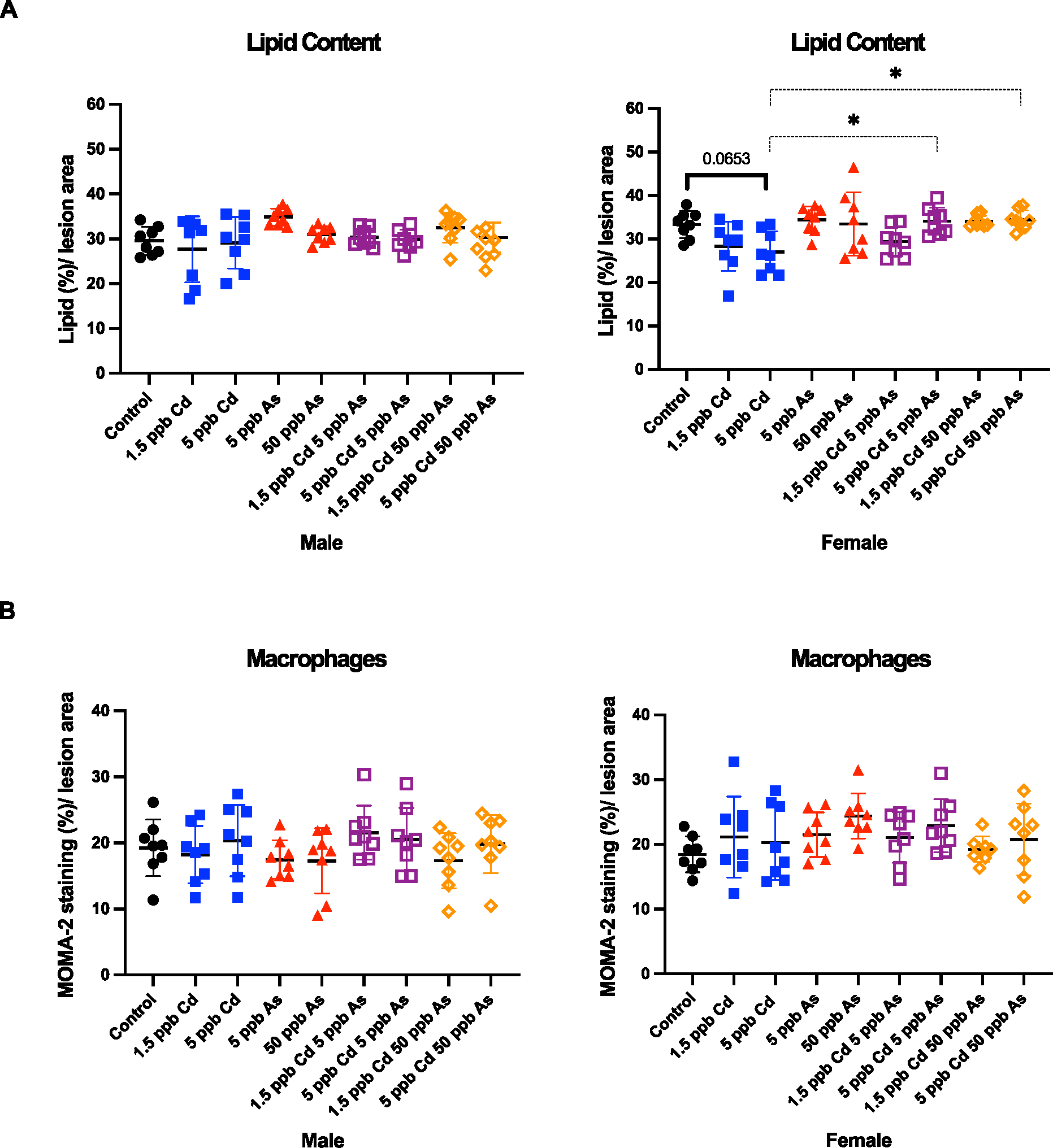
Few changes were observed in lipids and macrophages in plaques. No change in macrophage number in both sexes. Lipid content (A) and macrophage number (B) in the atherosclerotic plaque in the aortic sinus after low-dose arsenic, cadmium, and co-exposure in ApoE^−/−^mice. Plaque was quantified in the aortic sinus by oil red O (A) and MOMA-2 (B) staining using ImageJ. Each point represents a single mouse. Statistical significance is represented as follows: *p <. 05. (For interpretation of the references to colour in this figure legend, the reader is referred to the web version of this article.)

Previously, we reported that an exposure 10–200 ppb arsenic in ApoE−/−mice alters plaque composition towards a less stable phenotype with increased lipid content, decreased smooth muscle cells and decreased collagen content (Makhani et al., 2018). To assess the changes in plaque composition after low-dose metal exposure, we stained for these components, all of which play a role in plaque progression and regression. Here, we used oil red O staining to determine the change in lipid content in the plaque. In males, no significant changes were seen in lipid content. Interestingly, in females exposed to 5 ppb Cd, there was a decrease in lipid content, however, with the addition of 5 or 50 ppb arsenic, lipid levels were restored to control values (Fig. 4A, p < 0.05). Macrophages phagocytose lipids and become foam cells, which we assessed by MOMA-2 staining. In accordance with what we have observed historically (Makhani et al., 2018; Lemaire et al., 2011), no significant changes in macrophage numbers were observed across the exposure groups in both sexes (Fig. 4B), suggesting increased intracellular lipid retention in macrophages.

**Fig. 5.**
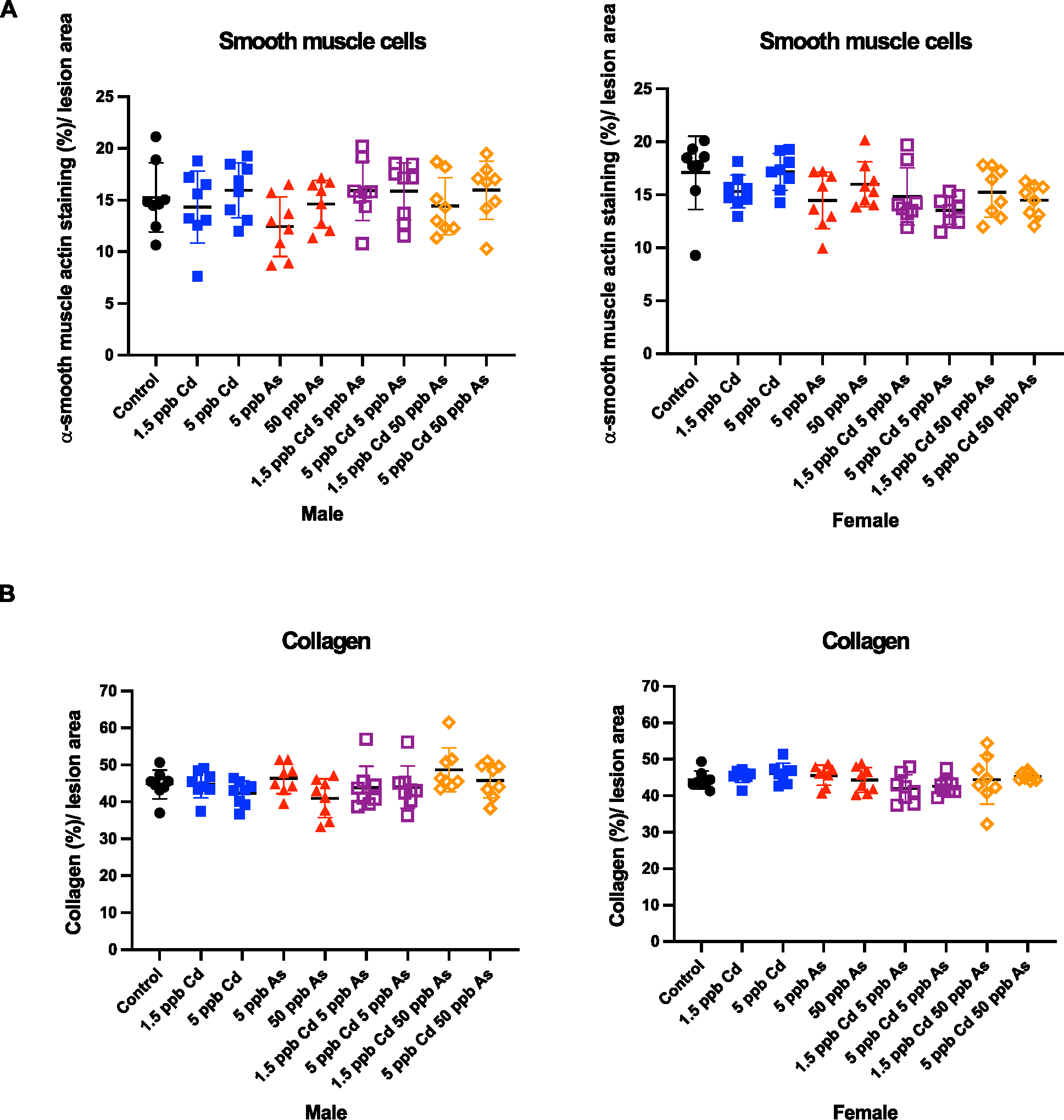
No changes were observed in smooth muscle cells and collagen. Smooth muscle cells (A) and collagen (B) content in the atherosclerotic plaque in aortic sinus after low-dose arsenic, cadmium and co-exposure in ApoE−/−mice. Plaque was quantified by *α*-smooth muscle actin (A) and picrosirius red (B) staining and imaged using ImageJ. Each point represents a single mouse. (For interpretation of the references to colour in this figure legend, the reader is referred to the web version of this article.)

**Fig. 6.**
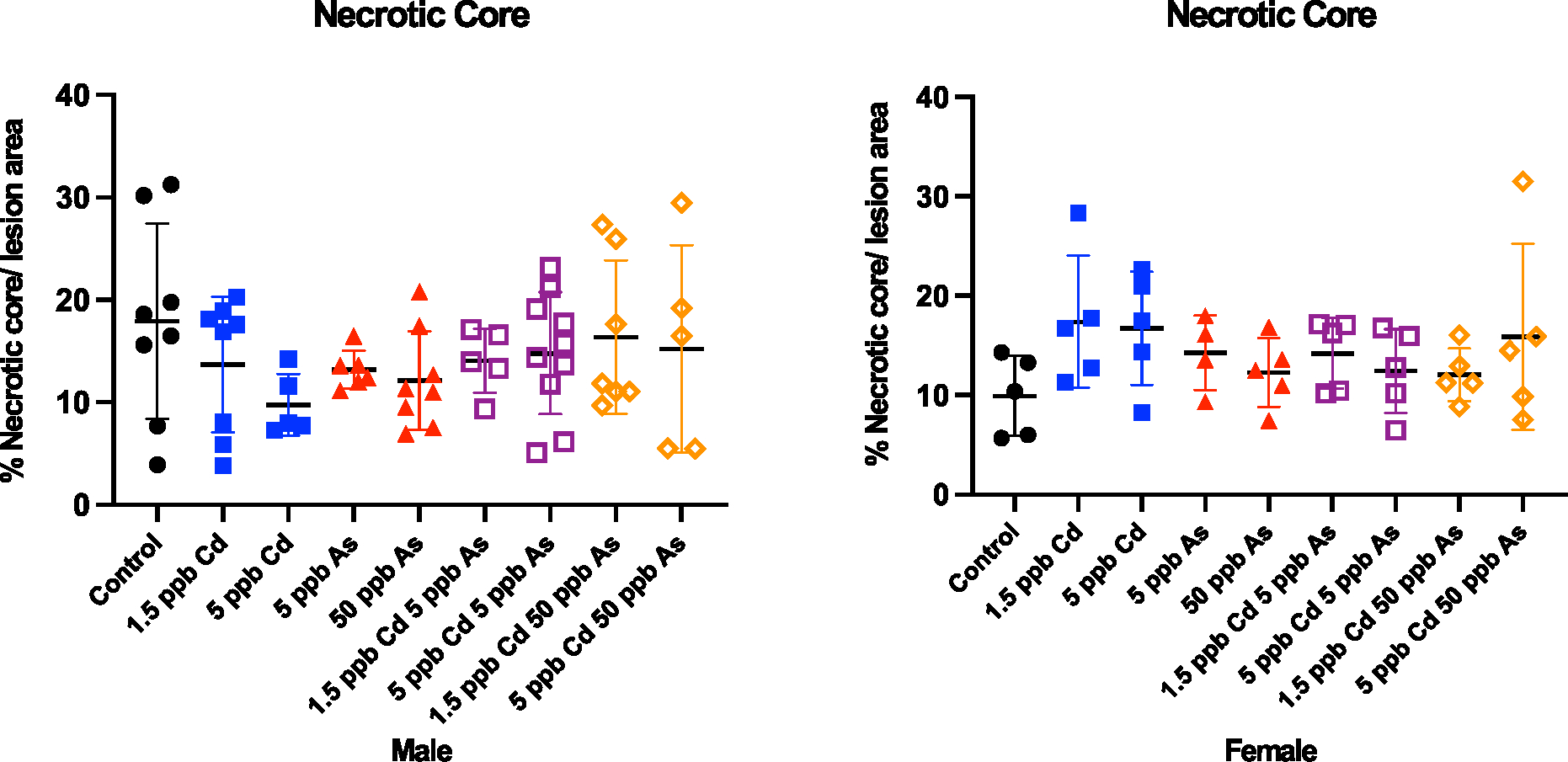
The size of the necrotic core in the atherosclerotic plaque did not change significantly in both male and female ApoE^−/−^mice after low-dose arsenic, cadmium, and co-exposures. Necrotic core was assessed from hematoxylin and eosin-stained aortic sinus sections and imaged using ImageJ. Each point represents a single mouse.

In atherogenesis, smooth muscle cells migrate, proliferate in lesions, and form a protective fibrous cap during plaque progression. In addition, smooth muscle cells synthesize extracellular matrix components, such as collagen, to increase plaque stability. We determined the percent smooth muscle cells in aortic sinus sections by α-actin staining and quantitated the collagen content by picrosirius red staining. Here, in both males and females, very few changes were seen in smooth muscle cell and collagen content (Fig. 5). In addition, we quantified the size of the necrotic core in the plaque by hematoxylin and eosin staining to assess plaque vulnerability induced by each metal and mixtures. In accordance with all the other findings, the size of the necrotic core did not significantly change (Fig. 6). Similarly, no significant changes were seen in the circulating lipid levels (total cholesterol, low-density lipoprotein, high-density lipoprotein, triglycerides) or glucose in males or females (Table S4). Thus, our *in vivo* studies demonstrate that the combinations of low-dose arsenic and Cd are not significantly worse than either metal alone with very few changes in plaque constituents.

## 4. Discussion

Together, our *in vitro* and *in vivo* studies suggest that low-dose metal mixtures of arsenic and Cd are not significantly more pro-atherogenic than either metal singularly. We found there is differential sensitivity to mixtures of arsenic and Cd in RAW 264.7 and C166 cells *in vitro*. Interestingly, mixtures of low-dose arsenic and Cd have synergistic interactions in macrophages, but antagonistic interactions in endothelial cells. Despite these differential interactions between arsenic and Cd, at such doses, these metals did not increase ROS in macrophages or adhesion molecule expression on endothelial cells as single agents or as mixtures *in vitro*. Previously done studies in RAW 264.7 cells with lower doses of Cd (10, 50 and 200 nM) have reported a dose-dependent and time dependent (24, 48 and 72 h) increase in lipid absorbance (Kumar et al., 2019). Here, at 0.05 μM (50 nM) Cd, we see an increase in lipid uptake at 24 h, although not statistically significant. In addition, exposure to higher concentrations of Cd decreased the rate of lipid uptake in RAW cells. More interestingly, higher concentrations of Cd suppressed arsenic-mediated lipid uptake in RAW 264.7 macrophages. *In vivo*, we see very few changes in plaque size and constituents in the ApoE^−/−^mouse model in both sexes. While in both sexes there are some increases in lesion size in both aortic arch (Fig. 3A) and aortic sinus (Fig. 3B), these changes do not exceed those caused by higher concentrations of arsenic or with high fat diet as previously observed (Lemaire et al., 2011; Makhani et al., 2018).

Previous *in vitro* studies have reported cell type-specific and dose-specific sensitivity to metal mixtures. In human keratinocytes, exposure to mixtures of arsenic, cadmium, chromium, and lead at low concentrations stimulated growth (Bae et al., 2001). However, with increasing metal concentrations, the combined toxicity changed from additive to synergistic cytotoxicity, followed by antagonistic interactions at the highest mixture concentration (Bae et al., 2001). Supporting the antagonistic effects at the highest dose, cellular defence mechanisms were also enhanced with increased levels of glutathione and metallothionein (Bae et al., 2001). Similar differential interactions with increasing metal concentrations were reported after exposure to arsenic, cadmium, mercury and lead in MCF 7 breast cancer cells (Klutse et al., 2009). These findings suggest that the nature of the interaction between metals and the combined toxicity may be concentration and cell dependent. In another study (Karri et al., 2018), the toxicity of lead, cadmium, arsenic, and methylmercury as single agents and as binary mixtures were assessed in HT-22 neuronal cells. In agreement to our findings in the endothelial cells, the cytotoxicity of the combination of arsenic and cadmium at low doses showed antagonistic effects in the neuronal cells (Karri et al., 2018). Thus, in addition to dose, cell type may also play a role in metal interactions in combined toxicity.

This study uses only murine cell lines, which could limit the breadth of applicability of the data. RAW 264.7 is a commonly used myeloid cell line shown to have stable macrophage-like phenotypic and functional characteristics until passage 30 (Taciak et al., 2018). All experiments with RAW 264.7 were carefully carried out with a passage number <30. Moreover, C166 cells are reported to exhibit normal endothelial characteristics. More importantly, they constitutively express murine VCAM-1 (Wang et al., 1996), therefore we chose to assess this adhesion molecule here amongst other pro-atherogenic cell surface markers. In addition to VCAM-1, VE-cadherin, a major determinant of endothelial cell contact integrity, (Vestweber, 2008) and caveolin-1 (Puddu et al., 2023) are other markers of endothelial cells involved in plaque progression. C166 cells do not express VE-cadherin or caveolin-1, therefore this was not studied here. However, previously done studies by Prozialeck and coworkers have reported that exposure to 100 nM (0.1 μM) Cd for 15 h caused a marked reduction in VE-cadherin at the cell-cell contacts in human umbilical vein endothelial cells (Prozialeck, 2000). With regards to arsenic exposure, at 24 h, a dose-dependent reductions in VE-cadherin was observed in human aortic endothelial cells (HAECs) at 1, 5 and 10 μM (Pereira et al., 2007). While this is interesting, we wanted to compare our *in vitro* studies with our mouse model hence we chose to work with murine cell line. Future studies including low-dose arsenic and cadmium exposure in a human cell line will be of interest.

Few studies have characterized the toxicological effects of arsenic and Cd as mixtures *in vivo.* Moreover, to our knowledge, this is the first study to investigate the pro-atherosclerotic outcomes in a mouse model after low-dose metal mixtures. In male rats, simultaneous intraperitoneal co-exposure to 10 mg/kg sodium arsenite and 2.6 mg/kg cadmium chloride altered the histopathology and the glutathione levels in various tissues produced by either metal alone. Interestingly, prior studies suggest that at a histopathological level, the acute toxicity of the combination is less toxic than cadmium alone in testes, whereas not readily apparent in liver and kidney (Díaz-Barriga et al., 1990). Thus, there is evidence of differential sensitivity to metals which differs across tissues. In relevance to cardiotoxicity, a follow-up study done in rats at the same doses showed that the combination of metals is more toxic in the heart tissue than either single metal (Yáñez et al., 1991). On a similar note, chronic exposure to the combination of 22.5 ppm arsenic in drinking water and 100 ppm Cd in diet exacerbated renal toxicity more than either metal alone in metallothionein-I/II null mice (Liu et al., 2000). These studies suggest that, in contrast to our findings, the mixture of arsenic and Cd is more toxic than the single metals. However, these studies were performed using higher doses and assessed different endpoints. Here, we were particularly interested in exposures at levels relevant to humans as defined based on approximate levels found in drinking water in the US population (Ravalli et al., 2022; Nigra et al., 2020). We also considered sex as a biological variable to extrapolate data to human populations. In both sexes at low doses, the combinations of arsenic and Cd are not worse than either metal singularly in the ApoE^−/−^mouse model. Together, our data show that low-dose exposure to mixtures of arsenic and Cd do not significantly increase atherosclerosis in a hyperlipidemic mouse model.

While the vascular system is a target of both arsenic and cadmium toxicity (Prozialeck et al., 2008), the lack of interaction *in vivo* may be explained by the differences in the biological fate and differences in pro-atherogenic mechanisms of both metals. Mammalian species have developed a mechanism to metabolize inorganic arsenic, where mice rapidly methylate and excrete arsenic. Cadmium bioaccumulates in mice (Tai et al., 2022) similar to humans. Thus, this may be a question of the concentrations utilized in our experimental design being too low. Perhaps at these low concentrations of Cd, chronic (>13 weeks) to near lifetime exposure may be needed to see significant Cd-induced proatherogenic effects alone or in combination with arsenic in a hyperlipidemic mouse model. Moreover, arsenic induces oxidative stress *via* production of ROS (Hu et al., 2020) whereas Cd competes with zinc, and binds to sulfhydryl groups which counteracts the antioxidant properties of glutathione, metallothionein and zinc superoxide dismutase (Bridges and Zalups, 2005; Lamas et al., 2023). The lack of cooperation by the metals is surprising, based on their non-overlapping mechanisms of ROS generation.

Our goal was to study human relevant concentrations of metal exposure in mice, however there are potential limitations to our *in vivo* study. First, we used a hyperlipidemic mouse model, although the mice were not fed a high-fat diet. Atherosclerotic lesions from animals fed the Western diet are more lipid-rich than regular diet (Getz and Reardon, 2006). Therefore, perhaps there may be pro-atherogenic effects after low-dose co-exposure to metal mixtures in the context of a Western diet. More importantly, mice metabolize inorganic arsenic much faster than humans. Mice are very efficient with arsenic methylation and have faster rates of urinary clearances of methylated metabolites (Vahter, 1999; Koller et al., 2020). Thus, equivalent exposures to human and mice may likely result in very different doses in both species. Considering the interspecies differences, it is possible that exposure to low-dose combinations of arsenic and Cd may produce more drastic effects on human tissue. Therefore, complementary human epidemiologic studies are needed to study mixtures of arsenic and Cd and risk of atherosclerotic-related outcomes at environmentally relevant concentrations of metal exposure.

## Supplementary Material

Supplement

## Data Availability

Data will be made available on request.
