# Supplementary material for: In vitro and in vivo approaches to assess atherosclerosis following exposure to low-dose mixtures of arsenic and cadmium
Source: Toxicol Appl Pharmacol. Author manuscript; Available in PMC 2024 Sep 20. (PMC11414205; doi:10.1016/j.taap.2023.116763)
Supplement: Supplement [file NIHMS2022942-supplement-Supplement.docx]

Supplemental Data for Subramaniam et al.

|  | **CADMIUM** |  |  |  |  |  |  |  |
| --- | --- | --- | --- | --- | --- | --- | --- | --- |
| **ARSENIC** | **0** $\boldsymbol{\mu M}$ | **0.01** $\boldsymbol{\mu M}$ | **0.05** $\boldsymbol{\mu M}$ | **0.5** $\boldsymbol{\mu M}$ | **1** $\boldsymbol{\mu M}$ | **2.5** $\boldsymbol{\mu M}$ | **5** $\boldsymbol{\mu M}$ | **10** $\boldsymbol{\mu M}$ |
| **0** $\boldsymbol{\mu M}$ | 100 | 102.866 ±  17.089 | 96.820 ± 3.085 | 92.333 ± 15.325 | 93.448 ± 41.788 | 86.350 ± 14.909 | 81.890 ± 18.126 | 74.583 ± 11.124 |
| **0.01** $\boldsymbol{\mu M}$ | 105.411 ± 13.021 | 82.883 ± 15.790 | 89.479 ± 14.861 | 81.518 ± 16.887 | 73.358 ± 18.278 | 70.029 ± 24.476 | 72.545 ± 15.897 | 64.028 ± 3.294 |
| **0.05** $\boldsymbol{\mu M}$ | 102.337 ± 6.951 | 89.161 ± 14.835 | 95.228 ± 25.070 | 78.419 ± 11.686 | 78.430 ± 11.242 | 68.894 ± 20.649 | 66.008 ± 12.320 | 68.673 ± 0.202 |
| **0.1** $\boldsymbol{\mu M}$ | 109.233 ± 10.967 | 95.132 ± 15.505 | 85.778 ± 18.487 | 71.638 ± 15.385 | 71.355 ± 15.941 | 70.868 ± 13.216 | 63.908 ± 14.186 | 62.093 ± 6.221 |
| **0.5** $\boldsymbol{\mu M}$ | 110.400 ± 13.208 | 85.748 ± 22.150 | 82.296 ± 9.476 | 79.750 ± 3.453 | 72.452 ± 10.719 | 70.247 ± 10.396 | 65.278 ± 10.598 | 71.255 ± 5.352 |
| **1** $\boldsymbol{\mu M}$ | 120.109 ± 12.545 | 89.454 ± 11.727 | 87.014 ± 23.339 | 78.967 ± 16.207 | 76.183 ± 13.203 | 66.269 ± 6.754 | 65.944 ± 9.169 | 66.974 ± 3.674 |
| **2.5** $\boldsymbol{\mu M}$ | 111.312 ± 6.648 | 78.586 ± 13.790 | 80.213 ± 27.077 | 78.786 ± 22.505 | 71.414 ± 17.875 | 61.389 ± 14.455 | 59.829 ± 6.286 | 67.655 ± 11.535 |
| **5** $\boldsymbol{\mu M}$ | 100.792 ± 15.271 | 79.651 ± 13.268 | 82.012 ± 23.095 | 80.307 ± 21.889 | 70.237 ± 22.281 | 66.419 ± 16.710 | 75.673 ± 9.649 | 66.217 ± 4.651 |

|  | **CADMIUM** |  |  |  |  |  |  |  |
| --- | --- | --- | --- | --- | --- | --- | --- | --- |
| **ARSENIC** | **0** $\boldsymbol{\mu M}$ | **0.01** $\boldsymbol{\mu M}$ | **0.05** $\boldsymbol{\mu M}$ | **0.5** $\boldsymbol{\mu M}$ | **1** $\boldsymbol{\mu M}$ | **2.5** $\boldsymbol{\mu M}$ | **5** $\boldsymbol{\mu M}$ | **10** $\boldsymbol{\mu M}$ |
| **0** $\boldsymbol{\mu M}$ | 100 | 97.778 ± 5.231 | 94.794 ± 2.699 | 92.933 ± 2.758 | 96.674 ± 7.217 | 98.162 ± 5.806 | 96.946 ± 10.285 | 97.723 ± 7.766 |
| **0.01** $\boldsymbol{\mu M}$ | 101.210 ± 5.228 | 96.067 ± 6.950 | 94.429 ± 6.612 | 93.965 ± 9.849 | 92.511 ± 10.088 | 97.125 ± 13.270 | 99.724 ± 11.945 | 99.612 ± 11.629 |
| **0.05** $\boldsymbol{\mu M}$ | 96.215 ± 2.374 | 87.798 ± 6.785 | 91.066 ± 10.608 | 91.824 ± 4.817 | 93.281 ± 2.187 | 95.618 ± 7.668 | 96.196 ± 11.779 | 94.202 ± 3.863 |
| **0.1** $\boldsymbol{\mu M}$ | 88.421 ± 5.285 | 91.770 ± 5.048 | 93.561 ± 9.734 | 87.538 ± 4.589 | 91.987 ± 12.815 | 86.554 ± 7.865 | 92.844 ± 14.542 | 92.367 ± 9.261 |
| **0.5** $\boldsymbol{\mu M}$ | 94.260 ± 2.599 | 89.123 ± 10.253 | 88.086 ± 9.481 | 84.665 ± 5.368 | 90.713 ± 18.069 | 91.791 ± 10.184 | 94.256 ± 11.232 | 96.629 ± 10.44 |
| **1** $\boldsymbol{\mu M}$ | 91.385 ± 5.617 | 86.125 ± 6.348 | 86.727 ± 6.924 | 86.219 ± 9.835 | 88.993 ± 11.444 | 88.474 ± 12.235 | 94. 315 ± 14.611 | 92.993 ± 7.032 |
| **2.5** $\boldsymbol{\mu M}$ | 88.465 ± 9.988 | 87.025 ± 10.779 | 85.537 ± 10.427 | 85.947 ± 9.658 | 84.441 ± 11.495 | 85.090 ± 11.805 | 89.978 ± 14.216 | 90.601 ± 13.605 |
| **5** $\boldsymbol{\mu M}$ | 89.290 ± 5.493 | 88.350 ± 7.797 | 85.741 ± 4.270 | 86.027 ± 2.728 | 86.943 ± 6.548 | 91.525 ± 8.436 | 90.650 ± 7.057 | 94.029 ± 5.371 |

Table S1. CellTiter-Glo Luminescent cell viability assay (n of 3) results showing the mean relative percent cell viability and standard deviation of RAW 264.7 murine macrophages (top) and C166 endothelial cells (bottom) after 24 hr exposure to combinations of arsenic and cadmium. No statistical significances were found between treatments in both cell lines.

|  | **CADMIUM** |  |  |  |  |  |  |  |
| --- | --- | --- | --- | --- | --- | --- | --- | --- |
| **ARSENIC** | **0** $\boldsymbol{\mu M}$ | **0.01** $\boldsymbol{\mu M}$ | **0.05** $\boldsymbol{\mu M}$ | **0.5** $\boldsymbol{\mu M}$ | **1** $\boldsymbol{\mu M}$ | **2.5** $\boldsymbol{\mu M}$ | **5** $\boldsymbol{\mu M}$ | **10** $\boldsymbol{\mu M}$ |
| **0** $\boldsymbol{\mu M}$ | 0 | 0 | 0 | 0 | 0 | 0 | 0 | 0 |
| **0.01** $\boldsymbol{\mu M}$ | 0 | 10.612 ± 20.205 | 5.612 ± 16.725 | 7.939 ± 10.448 | 6.799 ± 21.174 | 14.985 ± 23.055 | 8.163 ± 4.915 | 9.453 ± 10.994 |
| **0.05** $\boldsymbol{\mu M}$ | 0 | 4.624 ± 19.116 | 6.573 ± 17.309 | 11.113 ± 13.895 | 1.387 ± 22.150 | 16.118 ± 17.481 | 14.685 ± 7.359 | 4.665 ± 11.166 |
| **0.1** $\boldsymbol{\mu M}$ | 0 | 10.549 ± 17.944 | 17.129 ± 17.948 | 26.824 ± 21.088 | 24.274 ± 32.703 | 18.449 ± 14.241 | 24.013 ± 13.721 | 21.769 ± 20.486 |
| **0.5** $\boldsymbol{\mu M}$ | 0 | 7.781 ± 24.754 | 10.163 ± 9.639 | 7.608 ± 11.129 | 5.795 ± 20.184 | 11.339 ± 19.025 | 11.799 ± 14.891 | 1.809 ± 7.010 |
| **1** $\boldsymbol{\mu M}$ | 0 | 6.525 ± 18.541 | 12.106 ± 21.463 | 11.931 ± 29.941 | 5.000 ± 36.968 | 20.081 ± 18.624 | 15.946 ± 21.168 | 7.609 ± 16.357 |
| **2.5** $\boldsymbol{\mu M}$ | 0 | 16.549 ± 20.638 | 19.554 ± 24.342 | 12.640 ± 34.680 | 9.769 ± 18.624 | 24.961 ± 21.096 | 22.062 ± 21.168 | 6.928 ± 13.700 |
| **5** $\boldsymbol{\mu M}$ | 0 | 10.602 ± 18.977 | 12.471 ± 20.457 | 7.356 ± 31.456 | 6.547 ± 45.222 | 15.460 ± 29.841 | 1.510 ± 21.656 | 4.688 ± 22.168 |

|  | **CADMIUM** |  |  |  |  |  |  |  |
| --- | --- | --- | --- | --- | --- | --- | --- | --- |
| **ARSENIC** | **0** $\boldsymbol{\mu M}$ | **0.01** $\boldsymbol{\mu M}$ | **0.05** $\boldsymbol{\mu M}$ | **0.5** $\boldsymbol{\mu M}$ | **1** $\boldsymbol{\mu M}$ | **2.5** $\boldsymbol{\mu M}$ | **5** $\boldsymbol{\mu M}$ | **10** $\boldsymbol{\mu M}$ |
| **0** $\boldsymbol{\mu M}$ | 0 | 0 | 0 | 0 | 0 | 0 | 0 | 0 |
| **0.01** $\boldsymbol{\mu M}$ | 0 | (-0.619) ± 2.203 | (-1.033) ± 4.251 | (-2.168) ± 7.640 | 1.577 ± 11.258 | 1.319 ± 5.354 | (-2.300) ± 2.049 | (-1.356) ± 2.095 |
| **0.05** $\boldsymbol{\mu M}$ | 0 | 5.165 ± 4.741 | 1.023 ± 10.523 | (-2.382) ± 8.371 | (-1.430) ± 8.771 | (-1.181) ± 6.495 | (-2.385) ± 2.600 | (-2.112) ± 4.071 |
| **0.1** $\boldsymbol{\mu M}$ | 0 | (-8.636) ± 5.022 | (-10.775) ± 5.479 | (-8.319) ± 5.327 | (-8.335) ± 10.278 | (-1.547) ± 1.247 | (-6.815) ± 1.570 | (-8.086) ± 1.500 |
| **0.5** $\boldsymbol{\mu M}$ | 0 | 1.592 ± 5.358 | 0.961 ± 6.722 | 2.195 ± 3.088 | 1.899 ± 14.950 | 0.103 ± 6.366 | (-2.409) ± 1.329 | (-2.793) ± 0.927 |
| **1** $\boldsymbol{\mu M}$ | 0 | 2.257 ± 1.956 | (-0.052) ± 4.390 | (-1.290) ± 6.717 | (-1.820) ± 10.907 | (-0.035) ± 6.373 | (-5.290) ± 0.641 | (-5.405) ± 2.563 |
| **2.5** $\boldsymbol{\mu M}$ | 0 | (-1.361) ± 2.704 | (-1.626) ± 4.401 | (-3.767) ± 4.274 | (-0.147) ± 2.561 | (0.597) ± 3.069 | (-6.038) ± 2.869 | (-5.664) ± 1.730 |
| **5** $\boldsymbol{\mu M}$ | 0 | (-2.201) ± 10.253 | (-1.040) ± 9.817 | (-2.955) ± 4.672 | (-1.547) ± 9.907 | (-5.233) ± 7.459 | (-6.540) ± 5.397 | (-8.734) ± 5.887 |

Table S2. Excess over bliss analysis (n of 3) results showing the mean and standard deviation of RAW 264.7 murine macrophages (top) and C166 endothelial cells (bottom) after 24 hr exposure to combinations of arsenic and cadmium. No statistical significances were found between treatments in both cell lines.

Figure S1. Flow cytometry analysis of the expression of VCAM-1 on C166 cells after arsenic and cadmium treatment for 24 h (n of 3). No significant changes were observed in VCAM-1 expression after low dose metal treatments.

|  | **CADMIUM** |  |  |  |  |  |  |  |
| --- | --- | --- | --- | --- | --- | --- | --- | --- |
| **ARSENIC** | **0** $\boldsymbol{\mu M}$ | **0.01** $\boldsymbol{\mu M}$ | **0.05** $\boldsymbol{\mu M}$ | **0.5** $\boldsymbol{\mu M}$ | **1** $\boldsymbol{\mu M}$ |  |  |  |
| **0** $\boldsymbol{\mu M}$ | - 1. ± 0.27 | 1.84 ± 0.28 | 1.89 ± 0.23 | **0.91^a^**  ± 0.07 | **0.62^d^**  ± 0.16 |  |  |  |
| **0.01** $\boldsymbol{\mu M}$ | 1.73 ± 0.32 | 1.79 ± 0.22 | 1.88 ± 0.30 | **0.88^A,b^**  ± 0.08 | **0.67^B,c^**  ± 0.02 |  |  |  |
| **0.05** $\boldsymbol{\mu M}$ | 1.81 ± 0.51 | 1.79 ± 0.40 | 2.00 ± 0.37 | **0.99^a^**  ± 0.03 | **0.67^B,c^**  ± 0.04 |  |  |  |
| **0.1** $\boldsymbol{\mu M}$ | 1.59 ± 0.26 | 1.59 ± 0.23 | 1.78 ± 0.41 | 1.01 ± 0.03 | **0.73^A,c^**  ± 0.02 |  |  |  |
| **0.5** $\boldsymbol{\mu M}$ | 1.67 ± 0.28 | 1.55 ± 0.25 | 1.65 ± 0.31 | 1.12 ± 0.23 | **0.84^b^**  ± 0.14 |  |  |  |
| **1** $\boldsymbol{\mu M}$ | 1.52 ± 0.36 | 1.60 ± 0.26 | 1.62 ± 0.29 | 1.06 ± 0.11 | **0.83^b^**  ± 0.21 |  |  |  |
| **2.5** $\boldsymbol{\mu M}$ | 1.60 ± 0.27 | 1.60 ± 0.26 | 1.51 ± 0.27 | **0.95^a^**  ± 0.03 | **0.85^b^**  ± 0.15 |  |  |  |
| **5** $\boldsymbol{\mu M}$ | 1.60 ± 0.26 | 1.62 ± 0.35 | 1.67 ± 0.30 | 1.10 ± 0.15 | **0.89^b^**  ± 0.28 |  |  |  |

Table S3: Rate of Dil-OxLDL uptake per hour (mean and standard deviation) in RAW 264.7 cells after 24 h exposure to arsenic, cadmium and combinations. At higher concentrations of cadmium, the rate of Dil-oxLDL uptake was significantly reduced (represented in bold) alone and in combinations compared to cells only. In some of the combinations with higher dose of cadmium, the addition of cadmium significantly reduced the rate of lipid uptake compared to arsenic alone at the equivalent dose. A one-way ANOVA with a Tukey’s multiple comparison test was done. Statistical significance is represented as follows (compared to cells only): ^a^p < .05, ^b^p< .01, ^c^p< .001 and ^d^p< .0001 and ^A^p < .05, ^B^p< .01 (compared to arsenic only of same dose in combinations).

|  | **Total cholesterol (mg/dL)** | **LDL cholesterol (mg/dL)** | **HDL cholesterol**  **(mg/dL)** | **Triglycerides**  **(mg/dL)** | **Glucose**  **(mg/dL)** |
| --- | --- | --- | --- | --- | --- |
| **Control** | 622.88 ± 227.32 | 101.45 ± 37.21 | 32.32 ± 5.35 | 146.18 ± 64.76 | 294.06 ± 93.54 |
| **1.5 ppb Cd** | 438.38 ± 159.33 | 78.18 ± 54.00 | 40.62 ± 7.97 | 120.48 ± 54.05 | 232.90 ± 58.24 |
| **5 ppb Cd** | 507.04 ± 122.48 | 69.66 ± 18.89 | 32.02 ± 6.35 | 94.84 ± 27.19 | 264.16 ± 137.87 |
| **5 ppb As** | 416.45 ± 92.83 | 78.21 ± 25.06 | 37.63 ± 6.57 | 118.23 ± 13.08 | 240.75 ± 68.31 |
| **50 ppb As** | 481.22 ± 140.57 | 77.48 ± 28.93 | 34.74 ± 5.42 | 137.06 ± 57.12 | 256.30 ± 67.31 |
| **1.5 ppb Cd**  **5 ppb As** | 411.97 ± 189.33 | 74.70 ± 24.67 | 30.8 ± 0.10 | 112.13 ± 18.00 | 235.67 ± 47.47 |
| **5 ppb Cd**  **5 ppb As** | 436.78 ± 139.94 | 49.99 ± 13.60 | 34.48 ± 1.55 | 87.90 ± 24.00 | 252.08 ± 56.06 |
| **1.5 ppb Cd**  **50 ppb As** | 486.68 ± 149.76 | 69.69 ± 24.76 | 25.16 ± 12.15 | 91.16 ± 31.70 | 300.04 ± 71.54 |
| **5 ppb Cd**  **50 ppb As** | 515.58 ± 78.33 | 86.14 ± 32.77 | 37.14 ± 3.16 | 137.78 ± 30.45 | 290.82 ± 44.63 |

|  | **Total cholesterol (mg/dL)** | **LDL cholesterol (mg/dL)** | **HDL cholesterol**  **(mg/dL)** | **Triglycerides**  **(mg/dL)** | **Glucose**  **(mg/dL)** |
| --- | --- | --- | --- | --- | --- |
| **Control** | 476.88 ± 144.60 | 67.06 ± 12.77 | 38.03 ± 1.79 | 152.33 ± 60.76 | 316.33 ± 98.36 |
| **1.5 ppb Cd** | 249.50 ± 31.11 | 40.07 ± 5.77 | 23.05 ± 8.41 | 72.18 ± 28.20 | 224.43 ± 49.76 |
| **5 ppb Cd** | 328.22 ± 71.61 | 66.11 ± 14.86 | 28.64 ± 12.25 | 91.40 ± 10.85 | 228.84 ± 60.38 |
| **5 ppb As** | 446.22 ± 246.77 | 72.01 ± 48.10 | 20.44 ± 13.49 | 81.50 ± 39.79 | 236.22 ± 50.58 |
| **50 ppb As** | 618.54 ± 125.49 | 108.46 ± 51.22 | 36.38 ± 6.22 | 129.78 ± 26.90 | 305.98 ± 43.03 |
| **1.5 ppb Cd**  **5 ppb As** | 462.03 ± 37.17 | 74.58 ± 10.18 | 37.18 ± 4.48 | 112.48 ± 46.66 | 302.18 ± 96.72 |
| **5 ppb Cd**  **5 ppb As** | 471.80 ± 83.45 | 76.47 ± 18.40 | 40.78 ± 9.93 | 112.90 ± 49.74 | 295.65 ± 45.62 |
| **1.5 ppb Cd**  **50 ppb As** | 369.07 ± 75.78 | 67.78 ± 5.13 | 32.13 ± 1.03 | 76.27 ± 21.81 | 344.20 ± 63.02 |
| **5 ppb Cd**  **50 ppb As** | 486.40 ± 61.15 | 87.78 ± 13.40 | 39.44 ± 7.77 | 93.86 ± 11.87 | 284.46 ± 27.24 |

Table S4. No changes were observed in circulating lipids in males (top) and females (bottom): low density lipoprotein (LDL), high density lipoprotein (HDL), total cholesterol, triglycerides and glucose.
